# Extraction, Purification, and Component Identification of Monoterpene Glycosides from *Paeonia suffruticosa* Seed Meal

**DOI:** 10.3390/molecules28083498

**Published:** 2023-04-15

**Authors:** Fengqin Wang, Fuxia Hu, Zhenjia Zheng, Haoyan Zhao, Qitong An, Zhaosheng Wang

**Affiliations:** Key Laboratory of Food Processing Technology and Quality Control in Shandong Province, College of Food Science and Engineering, Shandong Agricultural University, Taian 271018, China

**Keywords:** *P. suffruticosa* seed meal, monoterpene glycoside, response surface, purification, identification

## Abstract

*Paeonia suffruticosa* (*P. suffruticosa*) seed meal is a byproduct of *P. suffruticosa* seed processing, which contains bioactive substances such as monoterpene glycosides, and has not been effectively utilized at present. In this study, monoterpene glycosides were extracted from *P. suffruticosa* seed meal using an ultrasound-assisted ethanol extraction process. The monoterpene glycoside extract was then purified by macroporous resin and identified using HPLC-Q-TOF-MS/MS. The results indicated the following optimal extraction conditions: ethanol concentration, 33%; ultrasound temperature, 55 °C; ultrasound power, 400 W; liquid–material ratio, 33:1; and ultrasound time, 44 min. Under these conditions, the yield of monoterpene glycosides was 121.03 mg/g. The purity of the monoterpene glycosides increased from 20.5% (crude extract) to 71.2% (purified extract) when using LSA-900C macroporous resin. Six monoterpene glycosides (oxy paeoniflorin, isomaltose paeoniflorin, albiflorin, 6′-O-β-D-glucopyranoside albiflorin, paeoniflorin, and Mudanpioside i) were identified from the extract using HPLC-Q-TOF-MS/MS. The main substances were albiflorin and paeoniflorin, and the contents were 15.24 mg/g and 14.12 mg/g, respectively. The results of this study can provide a theoretical basis for the effective utilization of *P. suffruticosa* seed meal.

## 1. Introduction

*Paeonia suffruticosa* (*P. suffruticosa*) is a plant of the Ranunculaceae and Paeonia families. It is native to Asia, Europe, and North America and is now widely cultivated in China and Japan [1]. Apart from its ornamental value, *P. suffruticosa* also has health benefits, such as anti-inflammatory and analgesic effects [2,3]. The seeds of *P. suffruticosa* are rich in oil and are often used to make seed oil. Since 2011, *P. suffruticosa* seed oil has emerged as a new food resource in China, and the planting of *P. suffruticosa* to obtain oil has increased rapidly [4]. Moreover, the annual output of *P. suffruticosa* seeds in China is as high as 2.4 × 10^5^ million kg, while the annual processing capacity of *P. suffruticosa* seed oil is about 20 million kg, resulting in a substantial quantity of seed meal. *P. suffruticosa* seed meal is a byproduct of edible oil production, accounting for 60% of seeds. However, most of the *P. suffruticosa* seed meal is discarded, except for a small amount used for processing animal feed [5,6]. If *P. suffruticosa* seed meal is not utilized for scientific purposes, it will result in a significant waste of resources and environmental pollution. Research indicates that the protein content of *P. suffruticosa* seed meal is 30% [7]. Additionally, *P. suffruticosa* seed meal contains monoterpene glycosides, flavonoids, and stilbenes, as well as other bioactive substances and trace elements, which have development and utilization value [8,9,10].

The seeds of *P. suffruticosa* contain characteristic monoterpene glycosides. These compounds are predominantly paeoniflorin as well as its analogues, including paeoniflorin, oxy paeoniflorin, albiflorin, and benzoyl paeoniflorin, among others [11]. Monoterpene glycosides can effectively alleviate different types of aches by regulating the nervous system [12]. Moreover, monoterpene glycosides can protect the nervous system by enhancing mitochondrial function [13]. They also have anti-inflammatory effects by inhibiting inflammatory mediators of lymphocytes and macrophages, and have anti-cancer effects by inducing the expression of apoptotic factors [14,15,16]. Monoterpene glycosides are potential pharmaceutical intermediates based on their diverse biological activities. Accordingly, it is of great importance to extract and purify monoterpene glycosides from *P. suffruticosa* seed meal. Water extraction and organic solvent extraction are commonly used for the extraction of monoterpene glycosides, whereas ultrasonic extraction has cavitation and mechanical effects that can increase the movement frequency and velocity of material molecules [17]. Ultrasound-assisted ethanol extraction can save time and solvent, increase the rate of leaching of the target substance, and be particularly suitable for the extraction of monoterpene glycosides. Macroporous resin is a porous polymer material and plays an important role in the selective adsorption of different substances; it is therefore widely used for the purification of natural drugs [18]. Consequently, it is feasible to purify monoterpene glycosides from *P. suffruticosa* seed meal using macroporous resin. In this study, the monoterpene glycosides from *P. suffruticosa* seed meal were extracted and purified, and the compounds were determined using liquid chromatography-mass spectrometry. The results of this study can provide a theoretical basis for the effective utilization of *P. suffruticosa* seed meal.

## 2. Results and Discussion

### 2.1. Extraction of Monoterpene Glycosides

#### 2.1.1. Single-Factor Analysis

**Figure 1:**
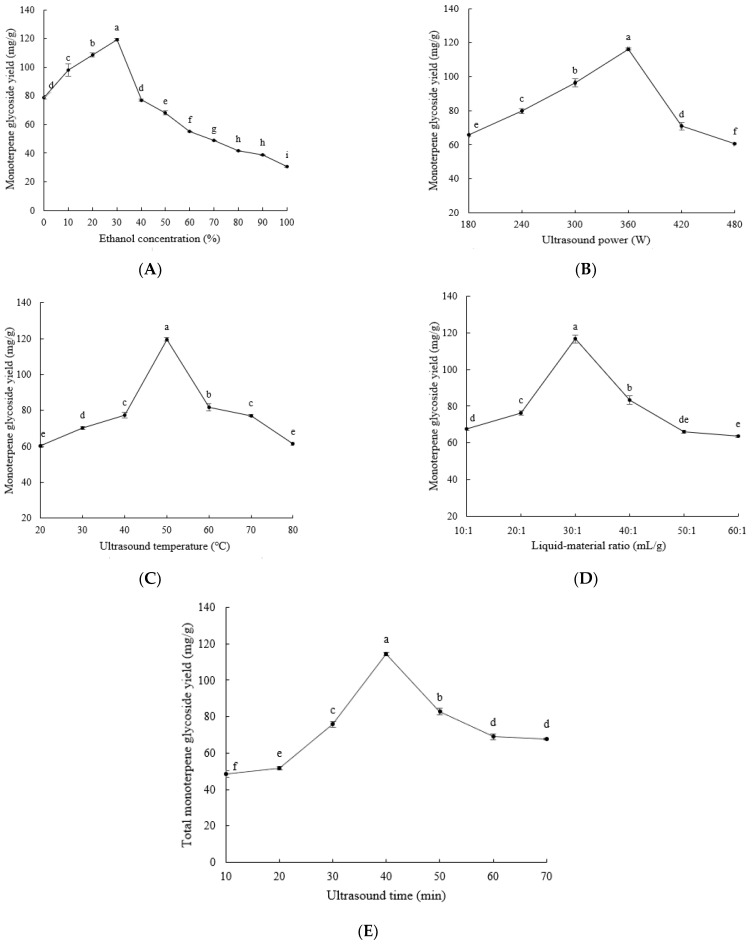
The effects of ethanol concentration (**A**), ultrasound power (**B**), ultrasound temperature (**C**), liquid–material ratio (**D**), and ultrasound time (**E**) on the extraction yield of monoterpene glycosides. Lowercase letters (a–i) indicate significant differences (*p* < 0.05).

The effects of ethanol concentration, ultrasound power, ultrasound temperature, liquid–material ratio, and ultrasound time on the extraction yield of monoterpene glycosides are shown in Figure 1. As shown in Figure 1A, with the increase of ethanol concentration, the extraction yield of monoterpene glycosides first showed an increasing trend, followed by a decreasing trend, peaking at 30%. These results may be due to the fact that the polarity of 30% ethanol was the closest to that of the monoterpene glycosides, which renders monoterpene glycosides more easily dissolvable, while a too low or too high polarity may not have been conducive to the extraction of monoterpene glycosides [19]. The results in Figure 1B show that the yield of monoterpene glycosides increased with the increase of ultrasound power, and peaked at 360 W. However, as the ultrasound power continues to increase, the thermal effect caused by the high-frequency vibration may destroy the structure of some monoterpene glycosides, decreasing the yield of monoterpene glycosides [20]. As shown in Figure 1C, when the ultrasound temperature was lower than 50 °C, the extraction yield of monoterpene glycosides increased with the increase in temperature, reaching the maximum when the ultrasound temperature reached 50 °C. This shows that within a certain range, an increase in temperature can accelerate the movement rate of molecules, reduce the surface tension of solvents, and increase the solubility of monoterpene glycosides in ethanol, thereby accelerating the dissolution of monoterpene glycosides. When the ultrasound temperature was over 50 °C, the extraction yield of monoterpene glycosides decreased with the increase in temperature, which could be attributed to the fact that the high temperature led to the destruction of the monoterpene glycoside structure. Figure 1D shows that as the liquid–material ratio increased, the extraction yield of monoterpene glycosides first increased and then decreased. When the liquid–material ratio reached 30:1, the extraction yield of monoterpene glycosides was the highest. These results may be due to the fact that an appropriate increase in the volume of ethanol could increase the contact area between the raw material and the extraction solvent, thereby promoting the dissolution of monoterpene glycosides. The cause of excessive ethanol reducing the extraction yield of monoterpene glycosides remains unclear. As shown in Figure 1E, when the extraction time was less than 40 min, the extraction yield of monoterpene glycosides from *P. suffruticosa* seed meal increased as time increased. This indicates that ultrasound may promote the dissolution of monoterpene glycosides from *P. suffruticosa* seed meal. However, when the extraction time exceeded 40 min, the yield of monoterpene glycosides gradually decreased as time increased. This indicates that when time is too long, some of the monoterpene glycoside structure may be destroyed [21].

#### 2.1.2. Optimization Using a Box-Behnken Design

Ethanol concentration, ultrasound temperature, ultrasound power, liquid–material ratio, and ultrasound time were used as variables, and were named A–E, respectively. The monoterpene glycoside yield (mg/g) of the extraction process was selected as the response value of the design experiments. Based on the results of the single-factor tests, a Box–Behnken experimental design of five factors and three levels was performed. The Box–Behnken design and results are presented in Table 1.

The analysis of variance (ANOVA) of Box–Behnken is shown in Table 2. The fitted model was as follows:

Y = 120.12 + 1.26A + 1.16B + 1.09C + 1.24D + 1.39E − 0.83AB − 0.27AC − 0.45AD − 0.29AE − 0.26BC − 0.025BD − 0.27BE − 0.26CD − 0.26CE − 0.20DE − 5.68A^2^ − 6.49B^2^ − 6.93C^2^ − 5.81D^2^ − 6.17E^2^

As shown in Table 2, the model’s F-value was 22.23, and the low probability value (*p* < 0.0001) indicated that the model was of great significance for the reasonable prediction of monoterpene glycoside production. In addition, the value of R^2^ was 0.9468, confirming that the response model could explain 94.68% of the total change. The lack of fitting *p*-value (0.0777) was higher than 0.05, indicating that the model had good reliability. Therefore, the response model was sufficient to reflect the expected optimization.

**Figure 2:**
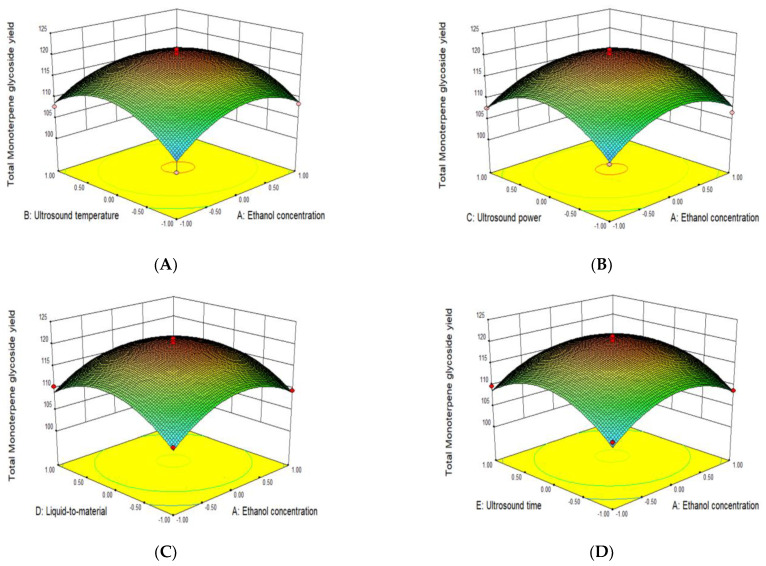

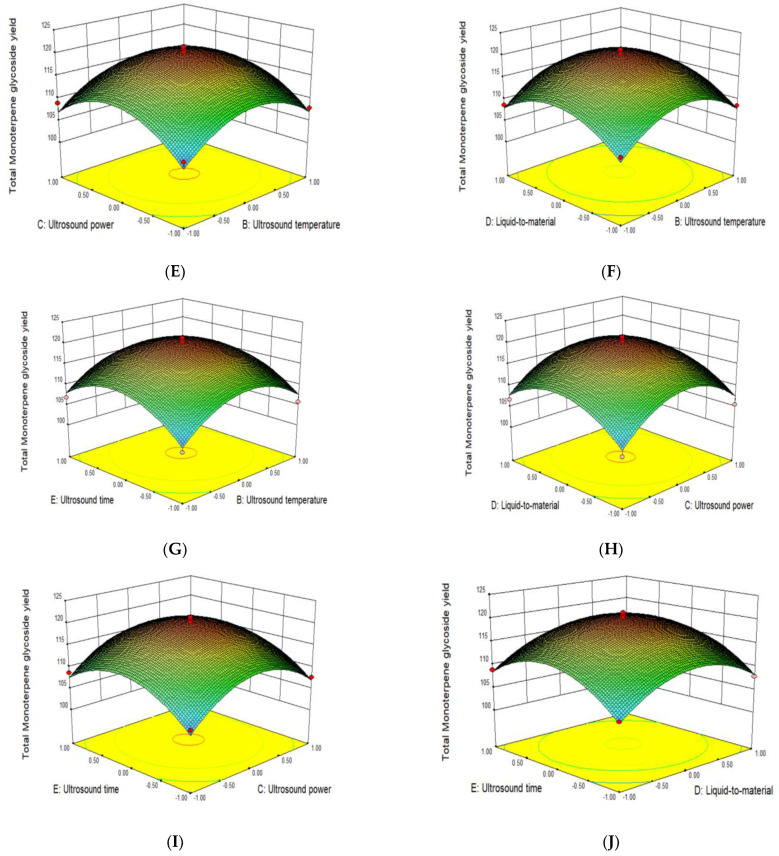
Interaction plots for ethanol concentration and ultrasound temperature (**A**); ethanol concentration and ultrasound power (**B**); ethanol concentration and liquid–material ratio (**C**); ethanol concentration and ultrasound time (**D**); ultrasound temperature and ultrasound power (**E**); ultrasound temperature and liquid–material ratio (**F**); ultrasound temperature and ultrasound time (**G**); ultrasound power and liquid–material ratio (**H**); ultrasound power and ultrasound time (**I**); and liquid-to-material ratio and ultrasound time (**J**).

The interactive effects of the five test variables on the monoterpene glycoside yield are shown in Figure 2. The steeper the slope of the response surface, the more significant the change of response value, with a flat slope indicating an unobvious effect [22]. Figure 2 shows that the interaction between the five factors was significant. Based on the above results, the optimal extraction conditions for the monoterpene glycosides from *P. suffruticosa* seed meal were as follows: ethanol concentration, 30.3%; ultrasound power, 388.8 W; ultrasound temperature, 53.5 °C; liquid–material ratio, 33:1 mL/g; and ultrasound time, 44.4 min. Under these conditions, the maximum predicted yield of monoterpene glycosides was 120.402 mg/g. Considering the practicality of the operation, ethanol concentration, ultrasound power, ultrasound temperature, liquid–material ratio, and ultrasound time were corrected to 30%, 400 W, 54 °C, 33:1 mL/g, and 44 min, respectively. In order to evaluate the effectiveness of the model, verification experiments were carried out under the best modification conditions. The actual yield of monoterpene glycosides was 121.03 mg/g, which was close to the predicted value, and the relative error was 0.52%. Therefore, these results confirmed the predictability of the model.

### 2.2. Purification of Monoterpene Glycosides

#### 2.2.1. Screening of Macroporous Resins

In order to select the most suitable macroporous resin for purifying the monoterpene glycoside extract from *P. suffruticosa* seed meal, we compared the adsorption and desorption performance of eight different macroporous resins for monoterpene glycosides. It can be seen from Table 3 that the adsorption capacity and adsorption ratio of LSA-900C, LSA-900E, and NKA-9 were the best, while the desorption ratio and recovery ratio of LSA-900C were the highest. This indicates that the polarity of LSA-900C macroporous resin was moderate; therefore, it is more suitable for the purification of monoterpene glycosides from *P. suffruticosa* seed meal.

#### 2.2.2. Static Adsorption and Desorption

**Figure 3:**
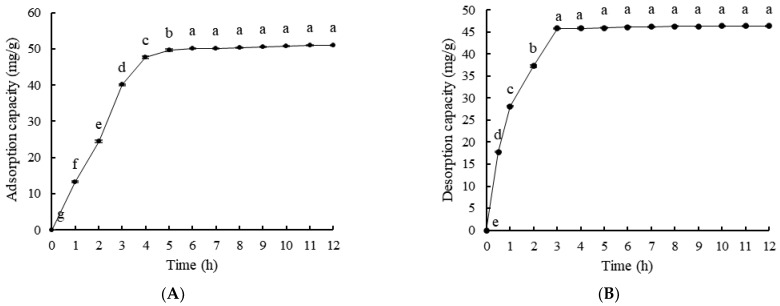
Kinetic curve of static adsorption (**A**) and desorption (**B**) of monoterpene glycoside extract from *P. suffruticosa* seed meal. Lowercase letters indicate significant differences (*p* < 0.05).

As shown in Figure 3A, when the adsorption time was 0–6 h, the adsorption capacity of LSA-900C macroporous resin for monoterpene glycosides from *P. suffruticosa* seed meal increased with the extension of time. The adsorption capacity was not significantly different after 6 h (*p* > 0.05), reaching adsorption equilibrium. As can be seen in Figure 3B, the desorption capacity of LSA-900C macroporous resin increased with the extension of time, and after 3 h, there was no significant difference in desorption capacity (*p* > 0.05). Therefore, the static adsorption time of LSA-900C macroporous resin for monoterpene glycosides from *P. suffruticosa* seed meal should be about 6 h, while the static desorption should be about 3 h.

**Figure 4:**
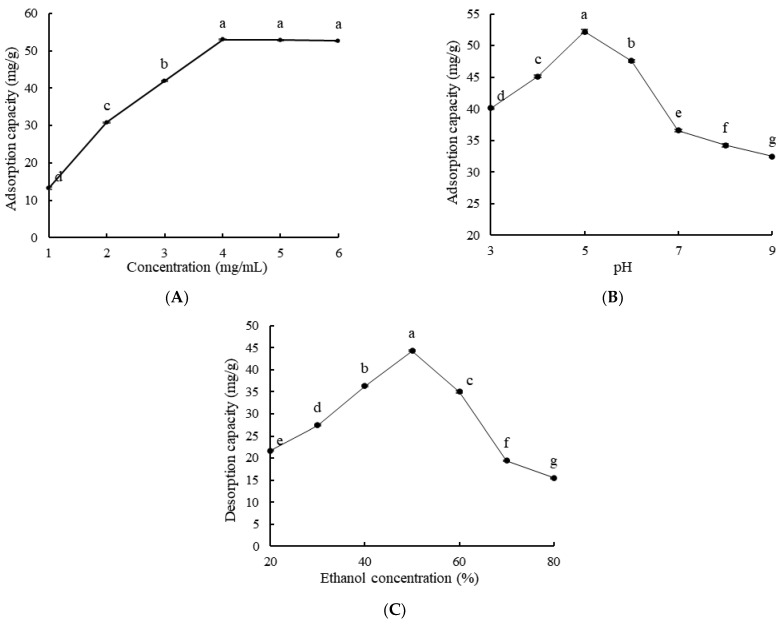
Effects of sample concentration (**A**), sample pH (**B**), and ethanol concentration (**C**) on the adsorption and desorption capacity of LSA-900C macroporous resin. Lowercase letters indicate significant differences (*p* < 0.05).

As shown in Figure 4A, the adsorption capacity of LSA-900C macroporous resin was the highest when the sample concentration was 4 mg/mL. When the concentration of monoterpene glycosides exceeded 4 mg/mL, there was no significant difference in the adsorption capacity of monoterpene glycosides (*p* > 0.05). If the concentration of monoterpene glycosides continued to increase, the extract would be wasted. As shown in Figure 4B, the adsorption capacity was the highest when the pH was 5. This could be due to the fact that different pH may affect the charge characteristics and ionization degree of the solution, thereby affecting the adsorption effect of macroporous resin. When pH < 5, the excessive acidity of the solution may reduce the solubility of monoterpene glycosides, making it prone to sedimentation. When pH > 5, the hydrogen bonding in the solution decreased, resulting in a decrease in adsorption capacity [23]. As shown in Figure 4C, the desorption capacity of macroporous resin increased as the ethanol concentration increased. This may be due to the destruction of hydrogen bonds formed between monoterpene glycosides extract and macroporous resin, and the gradual increase in the desorption capacity of LSA-900C macroporous resin. When the ethanol concentration was 50%, the desorption capacity of macroporous resin was the greatest; then, the desorption capacity gradually decreased as the ethanol concentration increased. This may be because the polarity of high concentration ethanol was small and the monoterpene glycosides were not easily eluted, thereby worsening the desorption effect. Therefore, the optimal conditions for static adsorption were as follows: sample concentration was 4 mg/mL, sample pH was 5, and eluent ethanol concentration was 50%.

#### 2.2.3. Dynamic Adsorption and Desorption of Macroporous Resin

**Figure 5:**
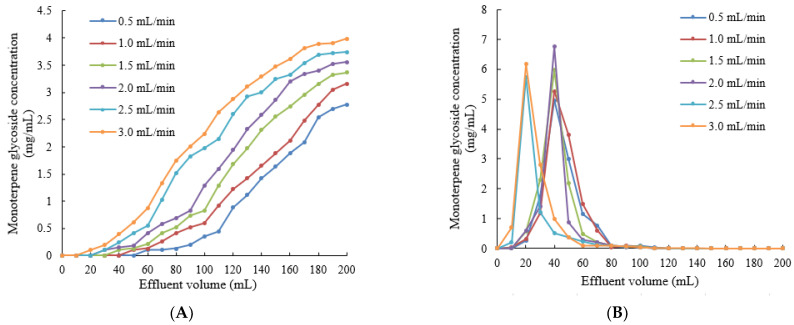
Effect of the sample solution flow rate on the adsorption of LSA-900C macroporous resin (**A**) and effect of the eluent flow rate on the desorption of LSA-900C macroporous resin (**B**).

When the concentration of monoterpene glycosides in the effluent reached 1/10 of the concentration of monoterpene glycosides in the sample solution, this was considered to be the leakage point [24]. As shown in Figure 5A, the concentration of monoterpene glycosides in the effluent increased as the sample volume increased. The lower the flow rate of the sample, the later the time of the leakage point. This may be due to the fact that monoterpene glycosides could make full contact with macroporous resin at the lower flow rate, which was conducive to the adsorption of LSA-900C macroporous resin for the monoterpene glycosides [25]. Under a high flow rate, the macroporous resin failed to fully adsorb the monoterpene glycosides and flowed out of the resin column, causing the leakage point to advance. A low sample flow rate would make the production cycle too long; therefore, the sample flow rate should be 1.5 mL/min and the sample volume should be 70 mL. The effect of the eluent flow rate on the desorption of LSA-900C macroporous resin is shown in Figure 5B. When the flow rate was 0.5–2.0 mL/min, the tailing phenomenon of the peak was more serious, which led to the use of more eluent. When the flow rate was 2.5 mL/min, the shape of the peak was concentrated and symmetrical. Therefore, the elution flow rate should be controlled at 2.5 mL/min and the volume of desorption solution should be 110 mL. When monoterpene glycosides were purified using LSA-900C macroporous resin under the optimal adsorption–desorption conditions, the purity of the monoterpene glycosides increased from 20.5% to 71.2%, which was 2.47 times higher. The results showed that LSA-900C macroporous resin had a good purification effect on monoterpene glycosides from *P. suffruticosa* seed meal.

### 2.3. Component Analysis of Purified Monoterpene Glycosides

**Figure 6:**
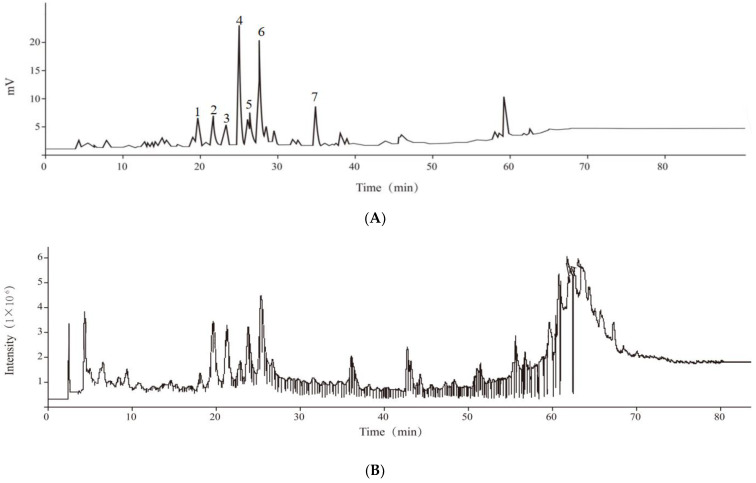

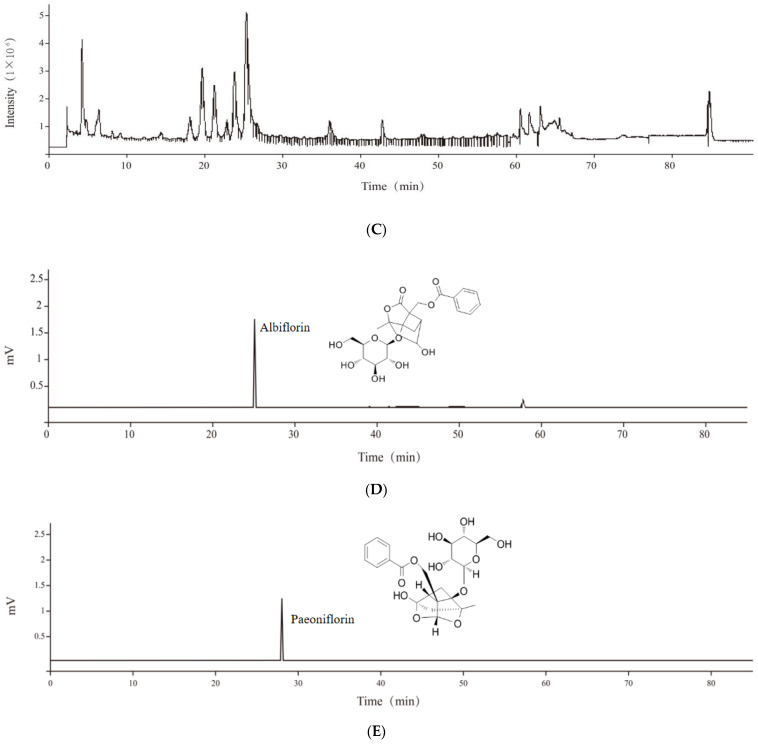
Analysis of the monoterpene glycoside compounds in the purified extract from *P. suffruticosa.* HPLC chromatograms (280 nm) of the monoterpene glycoside extract from *P. suffruticosa* (**A**) where: 1, unknown; 2, oxypaeoniflorin; 3, isomaltose paeoniflorin; 4, albiflorin; 5, 6′-O-β-D-glucoside albiflorin; 6, paeoniflorin; 7, paeonoside i. Total ion chromatograms (TICs) in positive (**B**), total ion chromatograms (TICs) in negative (**C**). HPLC chromatograms (280 nm) of albiflorin (**D**) and peoniflorin (**E**).

UPLC-Q-TOF-MS/MS was used to qualitatively analyze the compounds of the monoterpene glycoside extract from *P. suffruticosa* seed meal. As shown in Figure 6A, there were seven peaks (peaks 1–7) found in the HPLC chromatograms (280 nm) of the purified monoterpene glycoside extract from *P. suffruticosa* seed meal.

According to the chromatographic information corresponding to the second peak, [M + Na]^+^ was 519.4188, [M + K]^+^ was 535.1225, [M − H]^−^ was 495.1506, and the relative molecular mass of the compound was 496. Based on the study of the chemical composition of Moutan Cortex, it was speculated that the chemical substance corresponding to the second peak was oxypaeoniflorin [26]. According to the corresponding information of the third peak, [M + Na]^+^ was 665.2083, [M + K]^+^ was 681.1812, [M − H]^−^ was 641.2084, and the relative molecular mass of the compound was 642. Based on Deng’s study on the chemical constituents of Moutan Cortex, it was speculated that the compound was isomaltose paeoniflorin [27]. According to the chromatographic information of the fourth peak, [M + H]^+^ was 481.1721, [M + Na]^+^ was 503.1552, [M − H]^−^ was 479.1554, and the relative molecular mass of the compound was 480. Based on the chemical composition analysis of *Paeonia delavayi* var. *lutea* roots, it was speculated that the compound was albiflorin [28]. According to the chromatographic information of the fifth peak, [M + Na]^+^ was 665.2088, [M + K]^+^ was 681.1812, [M − H]^−^ was 641.2085, and the relative molecular mass of the compound was 642. Based on Wu’s chemical composition analysis of *P. suffruticosa* seed meal, it was speculated that the compound was 6′-*O*-β-d-glucoside albiflorin [29]. According to the chromatographic information of the sixth peak, [M + Na]^+^ was 503, [M + K]^+^ was 519.1287, [M − H]^−^ was 479.1554, and the relative molecular mass of the compound was speculated to be 480. According to the chromatographic information of the seventh peak, [M + Na]^+^ was 503.1545, [M + K]^+^ was 519.1363, [M − H]^−^ was 479.1555, and the relative molecular mass of the compound was speculated to be 480. Based on Li’s research, it was speculated that the sixth peak was paeoniflorin and the seventh peak was paeonoside i [28].

The fourth peak and the sixth peak were the main compounds in the monoterpene glycoside extract from *P. suffruticosa* seed meal. Therefore, the two main compounds were verified and quantified using HPLC. As shown in Figure 6D and E, the retention time of main peaks 4 and 6 in the HPLC chromatogram (λ 280 nm) of the purified monoterpene glycoside extract of *P. suffruticosa* seed meal was matched with the retention time of the albiflorin and paeoniflorin standards. This further confirmed that peak 4 was albiflorin and peak 6 was paeoniflorin, and the contents were 15.24 mg/g and 14.12 mg/g (*P. suffruticosa* seed meal), respectively.

## 3. Materials and Methods

### 3.1. Reagents and Materials

*P. suffruticosa* (*Paeonia suffruticosa* “Feng Dan”) seed meal was provided by Heze Huarui Oil Co. Ltd. (Heze, China), dried at 50 °C using a constant temperature drying oven (Supo Instrument Co. Ltd., Shaoxing, China), then pulverized and sieved (60 mesh). Ethanol was provided by Kaitong Chemical Reagent Co. Ltd. (Tianjin, China). Macroporous resin was obtained from Lanxiao Technology New Material Co. Ltd. (Xi’an, China). Methanol and formic acid were supplied by Oceanpak Alexative Chemical Co. Ltd. (Gothenburg, Sweden).

### 3.2. Extraction of Monoterpene Glycosides from P. suffruticosa Seed Meal

#### 3.2.1. Determination of Monoterpene Glycoside Concentration

The concentration of monoterpene glycosides in *P. suffruticosa* seed meal was calculated using spectrophotometry. A series of paeoniflorin standard solutions (5, 10, 15, 20, 25, 30, 35 μg/mL) was prepared with 50% ethanol as solvent. The absorbance of the standard solution at 230 nm was measured. The standard curve was drawn with the concentration of paeoniflorin standard solution x (μg/mL) as abscissa and absorbance y as ordinate. The regression equation was y = 0.0227x − 0.0014, R^2^ = 0.9998. The absorbance of the extract at 230 nm was measured, and the concentration of monoterpene glycosides in the extract was calculated according to the regression equation.

#### 3.2.2. Single-Factor Experiment Design

A quantity (1.0 g) of *P. suffruticosa* seed meal powder was accurately weighed and placed in a glass test tube with stopper (100 mL), and mixed with ethanol solution (concentration, 40%; 30 mL). It was then treated in an ultrasonic water bath at 40 °C and 300 W ultrasound power for 40 min with an ultrasonic processor (Ultrasonic Instrument Co. Ltd., Kunshan, China). After ultrasound treatment, the extract was filtered using a rapid filter paper (diameter of 11 cm), and the filtrate was collected in a 100 mL glass plug test tube. The concentration of monoterpene glycosides in the extract was determined following the method described in Section 3.2.1. The extraction yield of monoterpene glycosides was calculated according to the following equation:

Extraction yield of monoterpene glycosides (mg/g) = cv/m(1)

where: c, the concentration of the monoterpene glycosides in extract, mg/mL; v, the volume of extraction solvent, mL; m, the mass of *P. suffruticosa* seed meal powder, g.

According to the above test conditions, the single-factor tests were carried out by changing one of the factors: ethanol concentration (from 10% to 100%), ultrasound power (from 180 to 480 W), ultrasound temperature (from 20 to 80 °C), liquid–material ratio (from 10:1 to 60:1 mL/g) and ultrasound time (from 10 to 70 min). Setting ultrasound power, temperature, and time was realized by adjusting the button on the ultrasonic processor.

#### 3.2.3. Box–Behnken Design

On the basis of a single-factor experiment, according to the Box–Behnken method, a five-factor and three-level experimental design was used to optimize the extraction process of monoterpene glycosides from *P. suffruticosa* seed meal.

### 3.3. Purification of Monoterpene Glycosides

#### 3.3.1. Pretreatment of Macroporous Resin

The macroporous resin was immersed in anhydrous ethanol for 24 h, then washed with deionized water to no ethanol taste. After that, the resin was soaked in 5% HCl solution for 12 h, washed with deionized water to neutral, and then soaked in 5% NaOH solution for 12 h and washed with deionized water to neutral.

#### 3.3.2. Screening of Macroporous Resin

A quantity (1.0 g) of each type of macroporous resin (AB-8, NKA-9, HPD-100, LSA-900 C, LSA-900E, D-101, HPD-826, S-8, respectively) was accurately weighed and placed in a conical flask (50 mL) and mixed with the monoterpene glycoside crude extract from *P. suffruticosa* seed meal (concentration, 3 mg/mL; 25 mL), then placed in a water bath thermostatic oscillator (25 °C, 120 rpm) for 24 h. After the adsorption equilibrium was reached, the concentration of monoterpene glycoside in the crude extract was determined, and the macroporous resin was washed with deionized water until the absorbance of the effluent at 230 nm was close to 0. After the water on the surface of macroporous resin was sucked dry with filter paper, the microporous resin was loaded into a conical flask (50 mL) and mixed with 50% ethanol solution (25 mL). It was then placed in a constant temperature water bath shaker for desorption (25 °C, 120 pm) for 24 h. After reaching the desorption equilibrium, the concentration of monoterpene glycosides in ethanol solution was determined. The adsorption capacity, adsorption ratio, desorption ratio, and recovery ratio of each microporous resin were calculated according to the following equations:

Adsorption capacity (mg/g) = (c_0_v_0_ − c_1_v_1_)/m Adsorption ratio (%) = [(c_0_v_0_ − c_1_v_1_)/c_0_v_0_] × 100 Desorption ratio (%) = [c_2_v_2_/(c_0_v_0_ − c_1_v_1_)] × 100 Recovery ratio (%) = (c_2_v_2_/c_0_v_0_) × 100 (2)

where: c_0_, the initial concentration of the monoterpene glycosides of crude extract, mg/mL; c_1_, the concentration of the monoterpene glycosides of crude extract after adsorption, mg/mL; c_2_, the concentration of the monoterpene glycosides in ethanol solution after desorption, mg/mL; v_0_, the volume of crude extract, mL; v_1_, the volume of crude extract after adsorption, mL; v_2_, the volume of ethanol solution, mL; and m, the mass of the macroporous resin, g.

#### 3.3.3. Static Adsorption and Desorption Test

A quantity (1.0 g) of the selected macroporous resin and solution of monoterpene glycoside extract (concentration, 3 mg/mL; 25 mL) was added to a conical flask (50 mL) and placed in a thermostatic water bath shaker for oscillation adsorption (25 °C, 120 rpm) for 12 h. The supernatant (100 μL) was taken every hour to determine the concentration of monoterpene glycosides, and the static adsorption kinetic curve was drawn. After the adsorption equilibrium was reached, the macroporous resin was washed with deionized water until the absorbance of the effluent at 230 nm was close to 0. After the water on the surface of the macroporous resin was sucked dry with filter paper, the macroporous resin was placed in a conical flask (50 mL) and mixed with 50% ethanol (25 mL). It was then placed in a constant temperature water bath shaker for continuous shaking desorption (25 °C, 120 rpm) for 12 h. During this period, the supernatant (100 μL) was absorbed every hour to determine the content of monoterpene glycosides, and the desorption kinetic curve was drawn. The pH of crude extract was controlled at 4. The effect of the concentration of the crude extract on static adsorption was studied by changing the crude extract concentration from 1 mg/mL to 6 mg/mL. The concentration of crude extract was controlled at 3 mg/mL, and the effect of pH on static adsorption was studied by changing the crude extract pH from 3 to 9. The effect of eluent concentration on static desorption was studied by changing the eluent concentration from 20% to 80% under the static adsorption condition of 3 mg/mL and pH 4.

#### 3.3.4. Dynamic Adsorption and Desorption Test

The solution of crude monoterpene glycoside extract (concentration, 4 mg/mL; 200 mL) was placed in a 250 mL beaker and connected to a constant flow pump (Huxi Analytical Instrument Factory Co. Ltd., Shanghai, China) through the sample tube. The outflow tube of the constant flow pump was connected to the glass chromatographic column (Φ 1.6 cm × 50 cm), prefilled with pretreated macroporous resin (10 g). The effluent was collected using an automatic sample collector (Huxi Analytical Instrument Factory Co. Ltd., Shanghai, China). The sample flow rate was controlled by the constant current pump at 0.5, 1, 1.5, 2, 2.5, and 3.0 mL/min, respectively. The effluent was collected at 10 mL per tube using an automatic sample collector. The concentration of monoterpene glycosides in each tube was determined, and the dynamic adsorption curve was drawn. After adsorption, a large amount of deionized water was used to remove the impurities, and ethanol (concentration, 50%; 200 mL) was used to elute at a flow rate of 0.5, 1, 1.5, 2, 2.5, and 3 mL/min, respectively. The effluent was collected at 10 mL per tube using an automatic sample collector. The concentration of monoterpene glycosides in each tube was determined, and the dynamic desorption curve was drawn.

### 3.4. Identification of Monoterpene Glycosides from P. suffruticosa Seed Meal

#### 3.4.1. Preparation of Sample Solution

The monoterpene glycoside extract from *P. suffruticosa* seed meal was purified according to the determined purification process conditions. The eluent was freeze-dried after rotary evaporation to obtain the powder of monoterpene glycoside extract. The freeze-dried powder was dissolved in chromatographic methanol to prepare 2 mg/mL solution, which was passed through a 0.45 μm microporous organic filter membrane and placed in a 4 °C refrigerator for further experiments.

#### 3.4.2. Conditions of HPLC-Q-TOF-MS/MS

An Inertsil ODS-SP (5 μm, 4.6 × 250 mm) chromatographic column was used for analysis. Mobile phase conditions: A was 0.1% formic acid aqueous solution, B was chromatographic methanol; gradient elution conditions: 0–10 min, 15–20% B; 10–20 min, 20–30% B; 20–30 min, 30–40% B; 30–40 min, 40–60% B; 40–60 min, 60–100% B; 60–80 min, 100% B. The injection volume was 10 μL, the flow rate was 0.8 mL, and the column temperature was 30 °C. The detection wavelength was 280 nm.

Ion source: ESI; atomization pressure, 50 psi; fragmentation voltage, 90 eV; drying gas temperature, 200 °C; drying gas flow rate, 8 L/min; capillary voltage, 3500 V/3000 V; positive and negative ion mode scanning; *m*/*z* scanning range, 50–1200.

#### 3.4.3. Identification of Main Components by HPLC

The main preliminarily identified monoterpene glycosides were verified and determined using liquid chromatography. The mobile phase composition and gradient elution conditions were consistent with those described in Section 3.4.2.

### 3.5. Statistical Analysis

All data were measured at least 3 times for each sample. SPSS 26.0 was used for one-way analysis of variance, *p* < 0.05 was considered statistically significant.

## 4. Conclusions

In this study, monoterpene glycosides were extracted from *P. suffruticosa* seed meal using an ultrasound-assisted ethanol extraction process. The optimum extraction conditions were obtained, leading to an extraction yield of 121.03 mg/g. LSA-900C macroporous resin was selected for the purification of the extract, and optimal purification conditions were obtained, increasing the purity of the monoterpene glycosides 2.47 times. Finally, six monoterpene glycosides (oxypaeoniflorin, isomaltoside paeoniflorin, albiflorin, 6′-O-β-D-glucoside albiflorin, paeoniflorin, and paeonoside i) were found in the mass spectrometry information of the extract. These results provide a theoretical basis for the effective utilization of *P. suffruticosa* seed meal.

## Figures and Tables

**Table 1:** Box–Behnken design and results.

Run	A	B	C	D	E	Extraction Yield (mg/g)
1	1(40)	0(50)	0(360)	0(30:1)	1(50)	110.73 ± 0.32
2	0(30)	0(50)	0(360)	0(30:1)	0(40)	118.93 ± 0.84
3	0(30)	0(50)	1(420)	0(30:1)	−1(30)	107.57 ± 0.36
4	0(30)	0(50)	0(360)	−1(20:1)	−1(30)	105.62 ± 0.57
5	0(30)	0(50)	0(360)	0(30:1)	0(40)	120.16 ± 0.44
6	−1(20)	0(50)	0(360)	−1(20:1)	0(40)	106.32 ± 0.21
7	0(30)	0(50)	0(360)	0(30:1)	0(40)	120.81 ± 0.71
8	0(30)	1(60)	0(360)	0(30:1)	−1(30)	105.75 ± 0.44
9	0(30)	0(50)	−1(300)	−1(20:1)	0(40)	103.35 ± 0.21
10	0(30)	1(60)	0(360)	1(40:1)	0(40)	110.64 ± 0.42
11	0(30)	0(50)	0(360)	−1(20:1)	1(50)	108.91 ± 0.64
12	0(30)	−1(40)	0(360)	0(30:1)	−1(30)	103.54 ± 0.28
13	−1(20)	0(50)	0(360)	1(40:1)	0(40)	110.32 ± 0.16
14	0(30)	0(50)	−1(300)	1(40:1)	0(40)	106.71 ± 0.97
15	1(40)	0(50)	0(360)	−1(20:1)	0(40)	109.47 ± 0.61
16	0(30)	0(50)	−1(300)	0(30:1)	1(50)	108.69 ± 0.13
17	0(30)	0(50)	1(360)	−1(20:1)	0(40)	105.52 ± 0.29
18	0(30)	−1(40)	0(360)	−1(20:1)	0(40)	106.35 ± 0.23
19	0(30)	1(60)	−1(300)	0(30:1)	0(40)	107.84 ± 0.56
20	0(30)	−1(40)	1(420)	0(30:1)	0(40)	108.92 ± 0.50
21	0(30)	0(50)	1(420)	1(40:1)	0(40)	107.84 ± 0.66
22	0(30)	0(50)	0(360)	0(30:1)	0(40)	119.65 ± 0.42
23	0(30)	0(50)	0(360)	0(30:1)	0(40)	121.24 ± 0.13
24	1(40)	−1(40)	0(360)	0(30:1)	0(40)	108.46 ± 0.12
25	−1(20)	0(50)	−1(300)	0(30:1)	0(40)	104.34 ± 0.33
26	0(30)	−1(40)	0(360)	0(30:1)	1(50)	106.82 ± 0.74
27	−1(20)	1(60)	0(360)	0(30:1)	0(40)	107.79 ± 0.33
28	0(30)	1(60)	0(360)	0(30:1)	1(50)	107.93 ± 0.31
29	1(40)	0(50)	0(360)	0(30:1)	−1(30)	108.62 ± 0.12
30	1(40)	0(50)	−1(300)	0(30:1)	0(40)	106.53 ± 0.13
31	0(30)	1(60)	0(360)	−1(20:1)	0(40)	108.47 ± 0.41
32	−1(20)	0(50)	0(360)	0(30:1)	1(50)	109.68 ± 0.66
33	0(30)	0(50)	1(420)	0(30:1)	1(50)	109.84 ± 0.07
34	0(30)	1(60)	1(420)	0(30:1)	0(40)	110.04 ± 0.18
35	−1(20)	0(50)	1(420)	0(30:1)	0(40)	107.57 ± 0.28
36	0(30)	0(50)	−1(300)	0(30:1)	−1(30)	105.38 ± 0.14
37	1(40)	0(50)	1(360)	0(30:1)	0(40)	108.69 ± 0.48
38	−1(20)	−1(40)	0(360)	0(30:1)	0(40)	102.26 ± 0.15
39	0(30)	−1(40)	−1(300)	0(30:1)	0(40)	105.68 ± 0.33
40	0(30)	0(50)	0(360)	1(40:1)	−1(30)	107.83 ± 0.63
41	1(40)	1(60)	0(360)	0(30:1)	0(40)	110.68 ± 0.40
42	1(40)	0(50)	0(360)	1(40:1)	0(40)	111.65 ± 0.40
43	0(30)	0(50)	0(360)	0(30:1)	0(40)	119.94 ± 0.19
44	0(30)	−1(40)	0(360)	1(40:1)	0(40)	108.62 ± 0.44
45	0(30)	0(50)	0(360)	1(40:1)	1(50)	110.31 ± 0.28
46	−1(20)	0(50)	0(360)	0(30:1)	−1(30)	106.43 ± 0.12

**Table 2:** ANOVA of Box–Behnken.

ANOVA Source	Sum of Squares	df	Mean Square	F-Value	*p*-Value
Model	945.16	20	47.26	22.23	<0.0001
A	25.30	1	25.30	11.90	0.002
B	21.37	1	21.37	10.05	0.004
C	19.08	1	19.08	8.97	0.0061
D	24.78	1	24.78	11.65	0.0022
E	30.72	1	30.72	14.45	0.0008
AB	2.74	1	2.74	1.29	0.2671
AC	0.29	1	0.29	0.13	0.7168
AD	0.83	1	0.83	0.39	0.5382
AE	0.32	1	0.32	0.15	0.6992
BC	0.27	1	0.27	0.13	0.7244
BD	2.50 × 10^−3^	1	2.50 × 10^−3^	1.18 × 10^−3^	0.9729
BE	0.30	1	0.30	0.14	0.7092
CD	0.27	1	0.27	0.13	0.7244
CE	0.27	1	0.27	0.13	0.7244
DE	0.16	1	0.16	0.077	0.7835
A^2^	281.21	1	281.21	132.26	<0.0001
B^2^	367.48	1	367.48	172.84	<0.0001
C^2^	419	1	419	197.07	<0.0001
D^2^	294.66	1	294.66	138.59	<0.0001
E^2^	331.95	1	331.95	156.13	<0.0001
Residual	53.15	25	2.13		
Lack of Fit	49.75	20	2.49	3.66	0.0777
Pure Error	3.40	5	0.68		
Cor Total	998.31	45			
	R^2^ = 0.9468 R^2^_Adj_ = 0.9042 R^2^_Pred_ = 0.7957				

**Table 3:** Adsorption and desorption performance of different macroporous resins for monoterpene glycosides from *P. suffruticosa*.

Macroporous Resin	Adsorption Capacity (mg/g)	Adsorption Ratio (%)	Desorption Ratio (%)	Recovery Ratio (%)
AB-8	43.68 ± 0.44 ^bc^	58.24 ± 0.59 ^bc^	74.37 ± 1.39 ^d^	43.31 ± 0.45 ^c^
NKA-9	50.54 ± 1.38 ^a^	67.39 ± 1.85 ^a^	85.19 ± 1.15 ^b^	57.41 ± 1.72 ^b^
HPD-100	42.25 ± 0.22 ^cd^	56.33 ± 0.29 ^bcd^	61.42 ± 0.36 ^e^	34.60 ± 0.34 ^e^
LAS-900C	50.32 ± 0.50 ^a^	67.10 ± 0.66 ^a^	93.59 ± 1.28 ^a^	62.79 ± 0.45 ^a^
LSA-900E	49.99 ± 1.09 ^a^	66.66 ± 1.45 ^a^	85.69 ± 1.27 ^b^	57.11 ± 1.22 ^b^
D-101	44.42 ± 0.40 ^b^	59.22 ± 0.22 ^b^	55.29 ± 1.22 ^f^	32.74 ± 0.61 ^f^
HPD-826	37.18 ± 0.40 ^e^	49.57 ± 0.53 ^e^	81.25 ± 1.18 ^c^	40.27 ± 0.29 ^d^
S-8	40.92 ± 0.50 ^d^	54.57 ± 0.67 ^d^	23.05 ± 2.33 ^g^	12.57 ± 1.19 ^g^

Note: Lowercase letters indicate significant differences (*p* < 0.05).

## Data Availability

All data are available within the article.

## Sample Availability

Samples of the monoterpene glycoside extract from *P. suffruticosa* seed meal is available from the authors.
